# Supplementary material for: Yiai Fuzheng decoction inhibits triple-negative breast cancer by remodeling the immune microenvironment
Source: Front Immunol. 2025 Sep 30;16:1615631. doi: 10.3389/fimmu.2025.1615631 (PMC12518410; doi:10.3389/fimmu.2025.1615631)
Supplement: Supplementary file 2 [file DataSheet1.zip › Supplementary File 2/Treatment/C_vs_H/07_Pathway_Analysis/Pathway_Analysis/Sig_Pathways_Name/Cysteine and methionine metabolism.pdf]

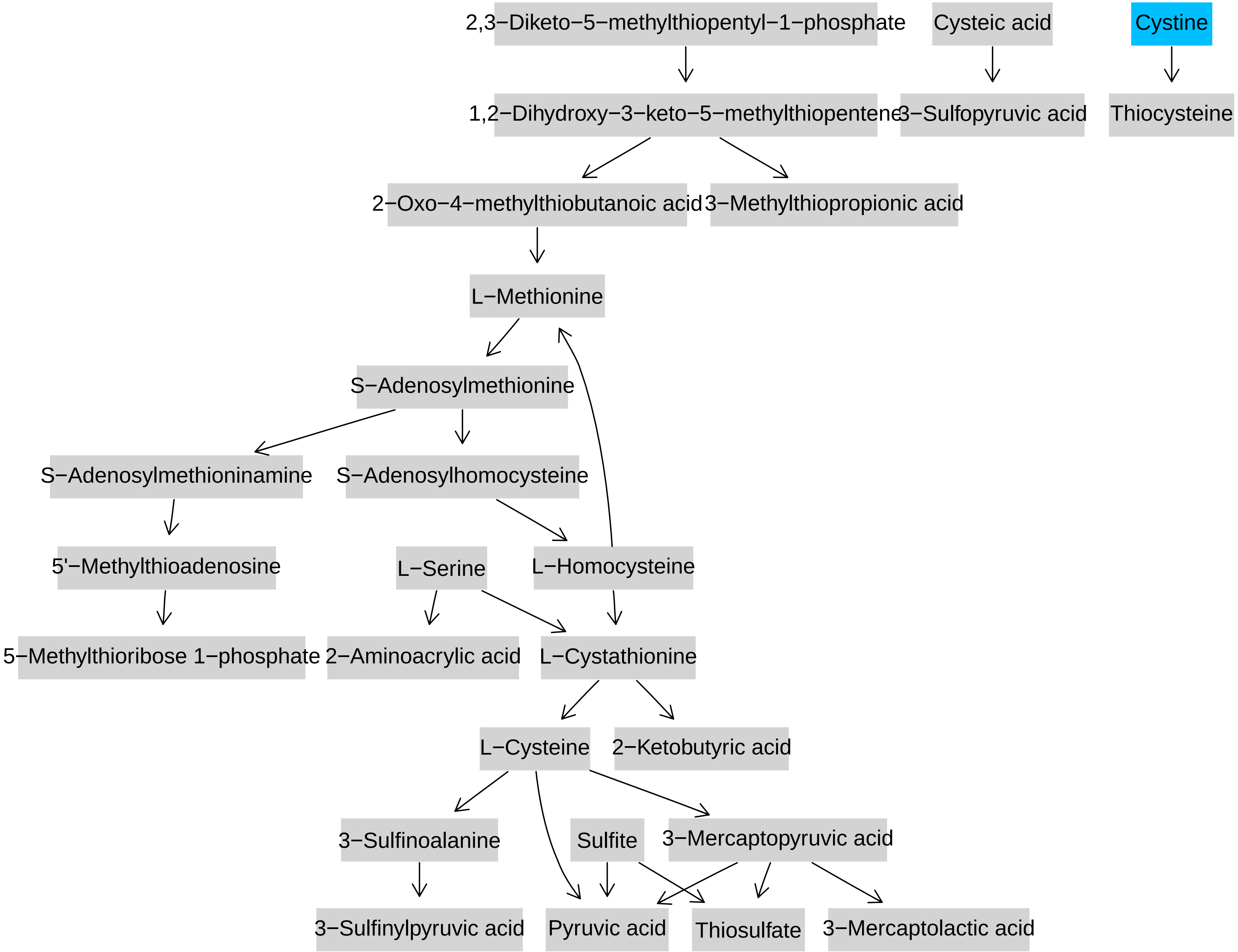

2,3−Diketo−5−methylthiopentyl−1−phosphate
Cysteic acid
Cystine
1,2−Dihydroxy−3−keto−5−methylthiopentene
3−Sulfopyruvic acid
Thiocysteine
2−Oxo−4−methylthiobutanoic acid
3−Methylthiopropionic acid
L−Methionine
S−Adenosylmethionine
S−Adenosylmethioninamine
S−Adenosylhomocysteine
5'−Methylthioadenosine
L−Serine
L−Homocysteine
5−Methylthioribose 1−phosphate
2−Aminoacrylic acid
L−Cystathionine
L−Cysteine
2−Ketobutyric acid
3−Sulfinoalanine
Sulfite
3−Mercaptopyruvic acid
3−Sulfinylpyruvic acid
Pyruvic acid
Thiosulfate
3−Mercaptolactic acid
